# Creating an eLearning Resource to Improve Knowledge and Understanding of Pregnancy in the Context of HIV Infection

**DOI:** 10.3390/ijerph111010504

**Published:** 2014-10-13

**Authors:** Carmel Kelly, Esther Reid, Maria Lohan, Fiona Alderdice, Dale Spence

**Affiliations:** 1 School of Nursing & Midwifery, Queen’s University Belfast, 97 Lisburn Road, Belfast BT9 7BL, UK; E-Mails: ereid04@qub.ac.uk (E.R.); m.lohan@qub.ac.uk (M.L.); f.a.alderdice@qub.ac.uk (F.A.); d.spence@qub.ac.uk (D.S.); 2 South Eastern Health & Social Care Trust, Newtownards Road, BT16 IRN, UK; 3 Southern Health & Social Care Trust, 68 Lurgan Road, BT35QQ, UK

**Keywords:** HIV, reproduction, knowledge transfer, eLearning

## Abstract

Patient narratives have much to teach healthcare professionals about the experience of living with a chronic condition. While the biomedical narrative of HIV treatment is hugely encouraging, the narrative of living with HIV continues to be overshadowed by a persuasive perception of stigma. This paper presents how we sought to translate the evidence from a qualitative study of the perspectives of HIV affected pregnant women and expectant fathers on the care they received, from the pre conception to post natal period, into educational material for maternity care practice. Narrative scripts were written based on the original research interviews, with care taken to reflect the key themes from the research. We explore the way in which the qualitative findings bring to life patient and partner experiences and what it means for nurses, midwives and doctors to be prepared to care for couples affected by HIV. In so doing, we challenge the inequity between the dominance of biomedical knowledge over understanding the patient experience in the preparation of health professionals to care for HIV affected women and men who are having a baby or seeking to have a baby.

## 1. Introduction

Evidenced based practice (EBP) has become central to the governance agenda for quality healthcare with one of the fundamental principles being that decisions about patient care should seek to integrate best available research evidence. However to achieve this goal, research cannot simply be disseminated into academic journals or otherwise, it must be re-packaged and tailored appropriately for specific audiences. In addition, more and more, the very existence of research has to be justified in terms of demonstrable benefits on health service policy, practice and patient care and thus requires careful thought about how this impact of research for end users can be developed. Knowledge transfer projects aim to take a targeted and tailored approach to sharing research evidence with knowledge users [1]. However, there is also inequity in what is considered to be valuable research evidence within the field of reproductive health and in health research more broadly. Quantitative research continues to be a dominant source of medical knowledge. Yet, increasingly efforts have been directed towards translating qualitative evidence into educational material for practice [2,3,4], as the insights generated by such research are considered fundamental to improving patients’ experience of healthcare [5]. In this paper we describe the development of our knowledge transfer project—“HIV & Pregnancy—Prepared to Care”—an eLearning educational resource which aims to prepare health care professionals to better understand the care needs of HIV affected women and men from reproductive decision-making to postnatal care. The aim of this paper is to describe the process of creating knowledge translation from qualitative research on HIV maternity care. In particular, we present the process of moving from formative research and its findings to the development of the eLearning product. In so doing, we address the inequity between the dominance of biomedical knowledge over understanding the patient experience in the preparation of health professionals to care for HIV affected women and men who are having a baby or seeking to have a baby.

The paper is structured as follows. We provide an overview of the original research. We then describe the various stages of the development of knowledge translation, including consultation and planning, video script development and eLearning resource production before drawing conclusions on the value of qualitative research as a source for knowledge translation and broader lessons to share for fellow academics seeking to develop skills for knowledge translation.

## 2. Stage 1: Formative Research

### 2.1. Methods

Following ethical approval from the Office of Research Ethics Committee for Northern Ireland, we conducted a longitudinal qualitative study between 2007 and 2010, which aimed to explore the real life contexts in which women and men, affected by a diagnosis of HIV, make reproductive decisions and their experience of pregnancy, childbirth and the care they received. The setting for this study was the regional centre for the management of HIV infection in Northern Ireland. In particular, three groups of women were selected for inclusion in the study: those who were aware of their diagnosis before becoming pregnant, those who learned of their diagnosis during antenatal screening and HIV negative women choosing a pregnancy with an HIV positive partner, along with a smaller group of fathers. The inclusion criteria for men was a HIV positive man in a sero-same or sero-different relationship whose partner was actively trying to conceive, was pregnant, or had recently given birth. Men whose female partners were participating in the study were excluded. All women and men who met the inclusion criteria were invited by their medical doctor to meet with the researcher for an introductory meeting to explain the study. Twenty nine in-depth interviews were conducted with ten women and five men at different stages in their journey through the reproductive trajectory. All five men were born in Africa, while the countries of birth for the women were Ireland (6), Africa (2), Eastern Europe (1) and Asia (1). The timing of the first interview depended on when the diagnosis of HIV occurred but the end point for interviews was in the postnatal period. In keeping with a narrative approach, the style of interview was unstructured and conversational. All but two interviews were conducted in participants’ homes. The analysis was multi-staged, at first focusing on each individual narrative and biographical transition between the interviews, second, a comparative thematic analysis to identify themes of commonality and difference across the interviews and third, an iterative analysis designed to go between emergent experiential themes from the study data and the wider body of work on HIV and pregnancy.

### 2.2. Results

The substantive findings from our study have previously been published in papers exploring three dominant overarching emergent themes, namely, the negotiation of risk in sexual relationships and reproductive decision making in couples when one partner is HIV positive and one is negative [6], the experience of testing HIV positive through routine antenatal screening [7], and the women’s experiences of pregnancy, maternity care and interactions with midwives [8]. In this section we summarize the overall key research findings, illustrating each finding with a direct quotation from the data. Later in the paper we will illustrate how these findings were translated into a script (Stage 3) for one of the vignettes in our resource.

HIV positive women’s desire to have a child reflects the cultural norm of motherhood as a natural desire and also a social expectation:

When I was diagnosed that was, obviously one of the first things that came into my mind was, “Is that it? Can I not have children now”? (Emma, HIV positive woman) 

For the HIV positive men, the prospect of fatherhood was central to rebuilding a sense of masculinity and wellbeing following diagnosis:

Having one woman, one wife and having healthy kids—these things affect me very much. When I think about it I am not useful anymore for people...I take the risk to do it because I want to feel as a man. (Henry, HIV positive man) 

Pregnancy signifies normality and the natural order to completing a committed relationship:

Then I got pregnant and I thought, maybe this is a good cover up. Because I didn’t think that I would have been able to get pregnant, so I am assuming that most people wouldn’t think that you can get pregnant if you are HIV positive. (Denise, HIV positive woman) 

Overwhelmingly, the decision to become pregnant is taken against a backdrop of increased confidence in the role of treatment in lengthening lives and protecting babies from infection:

We haven’t even thought, “god are we doing the right thing? What if we have a child with HIV?—because we just believe totally that there is only 1% (risk) and they can do so much now to prevent it”. (Emma, HIV positive woman) 

Sero-different couples negotiate HIV transmission risk within the context of their ongoing relationship. Love, commitment and a desire to conceive without medical interventions, alongside the added security of an undetectable viral load, significantly impact on men and women’s decisions to have unprotected sex in order to conceive. HIV positive women are more hesitant than men to take the risk of unprotected sex with their negative partner:

You know they suggested the various ways of how we could do it and we sat down and we discussed it and thought, ah well, seeing that I have been able to control the virus, and maintaining the viral load, we will just do it the normal way, without any interventions. (James, HIV positive man) 

A primary worry for both men and women was that their baby would not be born HIV positive, and therefore achieving an undetectable viral load in order to protect their children from HIV infection became a major goal of their pregnancy and childbirth experience:

I swallow 11 tablets a day just to make sure that he’s okay, you know, I really do hate it, and can’t wait to come off them….. anything just to make sure the baby is perfect, and yeah, I would’ve done it. (Claire, HIV positive woman) 

While women voiced confidence in the role of medical interventions to reduce onward transmission, they still worried about how they would personally respond to the medication (*i.e*., would they achieve undetectable viral load prior to delivery) and also about the possible negative effects the interventions might have for themselves and their babies:

I wondered what effect it would have on the baby and I think (partner) was also having the same feeling. He wanted me really to take them after being told what they would do, the safety for the baby. By then my feeling was they are too strong and I wonder what effect they will have to the baby, maybe abnormalities somewhere (Jennifer, HIV positive woman) 

Women accepted the need for interventions throughout their pregnancy and childbirth experiences as “being best for baby”:

The way I look at it, it’s bottle feeds, so that’s it. If I had experienced it the I probably would be saying, “oh what, I’m gonna to miss out”, but I’ve never experienced it so. (Emma, HIV positive woman) 

Significantly, one of women’s greatest worries was protecting against disclosure of their HIV status. As such, the medical management of their pregnancy required creating alternative stories and increased vigilance within the healthcare system:

I know I am going to be nervous when the time comes and I think I am worried more than I realise about keeping it a secret and how am I am going to explain having to go to Belfast, for the treatment or for the birth even. (Denise, HIV positive woman) 

Stigma, whether real or perceived, continues to dominate the symbolic significance of HIV. Women’s and men’s interactions with healthcare professionals can either contribute to their feelings of ‘normality’ or sharpen their experience of difference:

I saw that she wanted to let the other nurse know that I am positive but it was like (gestures whispering), so I said to her, “you can say it out loud”, you know, because it was upsetting to me when you see that she is trying to do something that I should not see but I can. (Anna, HIV positive woman) 

Our study findings revealed how the symbolic significance of HIV as a stigmatising condition was sometimes greater than the men’s and women’s concerns for the medical management of their HIV during pregnancy, the focus of which was reducing the risk of HIV transmission to their baby. While the women and men recounted an overall positive experience of care, significantly, every participant could also recount a negative experience of care. Lack of knowledge and experience among caregivers, breaches of confidentiality and the experience or perception of being treated differently because of their HIV status were central to participants’ negative experiences of care. Pregnancy and parenthood symbolised normality, and yet, the pervasive presence of stigma threatened the experience of pregnancy and care, requiring particular attention to the management of information.

## 3. Stage 2: eLearning Resource Development: Planning and Consultations

### 3.1. Methods

The research team worked with three consultation groups to inform the process of resource development. First, a group of HIV positive women were recruited via a local non statutory support organization, Positive Life. Second, two groups of healthcare professionals including representatives from obstetrics, midwifery and social services were invited to inform project development. The first, a professional users group reflected the intended end users, while the second, a project steering group included professionals who were strategically placed to support the delivery of the resource as part of continuing professional development training for maternity care staff across all of our health and social care trusts. These groups convened at three times throughout the project’s development phase. Initial discussions between the project team and consultation groups focused on the ultimate goals and uses of the product we wanted to produce. Between six and eight months into the project, and prior to the filming of vignettes, the consultation groups met again to focus on the scripts for authenticity and potential for learning. Our final meeting with the consultation groups was to pilot the first resource production. Close scrutiny of the usability of the interactive features of the resource occurred at this stage and modifications were made by the tool developer prior to final production.

### 3.2. Results

We agreed that our aim was to design and develop an interactive eLearning resource incorporating research informed patient narratives which: (a) presented both biomedical and qualitative research; (b) was modular to reflect the reproductive journey; (c) could be used as a quick reference or to promote reflective practice; (d) provided accessible links to evidence based medicine and further support; and (e) could be accessed individually or used in a classroom to promote discussion. Throughout the resource, evidence based information and guidance on HIV management [9] would be presented alongside interviews with key healthcare professionals and the scripted vignettes presented by actors. The scripts for the vignettes (Stage 3), written by the research team, were based on the original research interviews, with care taken to reflect the key themes from the research. The process of development is described in greater detail below. The decision to use actors reflected the difficulty in real life for many HIV positive people to have their voice heard. However, one HIV positive woman, who was part of the consultation group, chose to tell her own story, opting to be filmed in silhouette to protect her identity. Given the time pressures on staff working within the health sector, it was also agreed to offer users the option of a short course of two modules or a longer course of six modules. The content of each module was agreed and is outlined in Table 1. Our aim was that completion of the short course would only require one hour, while the full course would take approximately three hours to complete. It was also considered important that following a short self- assessment questionnaire, users should have the opportunity to print a certificate of completion for their professional portfolio.

**Table 1:** Outline of the six modules in the eLearning resource.

Module Title	Module Content
Module 1: HIV awareness	What is HIV? Key statistics and milestones Transmission risks Pregnancy and HIV
Module 2: Living with HIV	Interactions with healthcare professionals Confidentiality Stigma Culturally sensitive care Fatherhood and HIV
Module 3: Choices and risks for conception	When the man is HIV positive When the women is HIV positive All couples affected by HIV
Module 4: Diagnosing HIV during pregnancy	Antenatal screening Results Psychological support following diagnosis
Module 5: HIV care during pregnancy	ARV and adherence Monitoring HIV Impact of HIV on pregnancy
Module 6: Maternity care in the context of HIV	Obstetric management Neonatal management Breast feeding Experience of delivery

## 4. Stage 3: eLearning Resource Development: From Research Themes to Video Scripts

### 4.1. Methods

As one of the key aims of our eLearning resource was to influence perspectives, challenge assumptions about living with HIV and to reduce the social distance shaped by stigma which creates a “them” and “us” divide between health professionals and patients, the purpose of the videos was to present a human face to the experiences uncovered in our research [10]. While previous academic endeavours, most notably the Oxford University *Healthtalkonline* project, mostly presents films of peoples’ real life experiences of health related issues (http://www.healthtalkonline), our approach to develop vignettes from our original research interviews afforded us the opportunity to protect the anonymity of our participants’ and also to develop a composite of many participants experiences which represented our interpretations following data analysis [11].

The initial idea to develop vignettes came from the analysis of the interviews with four women in our study who had all received their HIV diagnosis as part of routine antenatal screening, from which we developed a composite narrative. This was a collective testimonial drawing on all of the women’s experiences. The vignette was developed to capture the essence of the disruption to life caused by a diagnosis of HIV during pregnancy [7]. The power of a narrative vignette to produce an empathic response in an audience was highlighted to us when we worked with a drama student to record this vignette as a method for presenting our empirical material at conferences.

We set out to develop and record eight composite narratives which reflected the perspectives of both women and men in both sero-same (both HIV positive) and sero-different (one HIV positive and one HIV negative) relationships. Our initial idea was that each short vignette would complement the written information in each module. The scripts for the vignettes were developed by an iterative approach of moving between the research themes, the original interview transcripts and our wider understanding of both the medical and social literature pertaining to HIV and reproduction. As described by Sandleowski *et al.* [2], as researchers, we were most interested in ‘ensuring fidelity of the script to the findings’ and of emphasising the social rather than medical aspects of HIV infection. However, as nurses and midwives we also had a professional responsibility to ensure accurate and evidence based medical information. Similar to Blickem and Priyadharshini [11] our vignettes were thus a composite of many stories and therefore, “were neither fully fact nor fully fiction but situated in the realm of plausibility” (624). In addition, the interviews with health care professionals, which were not scripted, allowed us to achieve this balance. For example, in the script illustrated below, both Kate and John, as a sero-different couple talk about their decision to have unprotected sex in order to conceive, based on the evidence that a person is not sexually infectious with an undetectable viral load for six months or more and no other concomitant sexually transmitted infection [12,13]. However, in practice, clinicians find it problematic to advise sero-different couples that transmission risk is zero, so in the interview with our HIV physician, Say Quah acknowledges that while this is what many couples choose to do, he qualifies this with a statement that this advice is not in the recommended guidelines.

### 4.2. Results

In this section, we have taken the example of our longest video to illustrate the result of the process of developing a video script from our research findings. On the advice of the professional users group, the longest video, tells the ‘whole story’ of Kate, who is HIV positive, and, John, who is HIV negative, through their journey from reproductive decision-making to after the birth of their baby. It is interspersed with interviews with our HIV physician and obstetrician. This video sits within Module 1, the idea being that if a user only completes the first module, then this video incorporates the key qualitative research themes from our findings (Stage 1) alongside a key biomedical message from the healthcare professionals: *With an undetectable Viral Load at 36 weeks, the woman’s management during labour and delivery should follow the same guidelines as for any other pregnant woman.* This video can be viewed at www.qub.ac.uk/hivandpregnancy (Module 1: Pregnancy and HIV).

The script for Kate & John’s vignette:

**Kate:** Like most women, I always wanted to have a baby, but thought it wasn’t an option anymore when I was diagnosed with HIV. I felt bad for my partner who is HIV negative. Sometimes I felt he was denied being a father because of me, and I knew he’d make a great dad. Family and friends were always hinting at why we hadn’t started a family, but we laughed it off, it was our little secret. However, over the years, with improvements in treatment and people with HIV living longer, it started to feel possible. The doctors told us that the risk of the baby having HIV has gone down to 1%. 

HIV physician 

**Kate:** We talked about all the ways I could possibly conceive because obviously I wanted to avoid passing the virus on to my partner. The doctor advised a syringe but it seemed so clinical. In the end we decided that because my viral load has always been well controlled, we would try and conceive naturally, having unprotected sex, but only when I was ovulating. But even though the doctors said the risk was low, it was still a worry. 

**John:** I had just resigned myself to never having a baby, Kate’s health was always the most important thing. We read all the leaflets, researched on the internet, spent a lot of time with the HIV consultant asking lots of questions and he spelt it all out for us. It gave us new hope that we could be like other couples and have a baby together, and so, I was happy to take the risk, small as it was. It felt strange at first, you know, having unprotected sex after years of being so careful. I know the doctor would have preferred we used a syringe as he said there was still a risk of transmission from unprotected sex. But I had weighed it all up and I thought the time was right and it was worth it, you know...coming from a time you thought you’d never have children to now, being a dad, it’s just been really special! 

HIV physician 

**Kate:** We were so thrilled when I got pregnant. Sometimes you can actually forget that you are HIV positive, but then you have to take the tablets every day and that reminds you. I was a bit nervous, you know about side effects and that, but when you see your viral load test staying so low, you know you are doing the best for your baby. John was so supportive…making me take my tablets everyday and coming with me to all my appointments, making sure I kept healthy. The staff at the hospital were great too, making me feel like any other pregnant woman. They told me that if my viral load was undetectable at 36 weeks I could even have a normal vaginal delivery, so it was worth it, I would have done anything to make sure my baby was alright! 

Obstetrician 

**Kate:** I had planned to have a normal birth but as the time got closer I got nervous. If I’m honest I was more worried about going into labour early and ending up in our local hospital and then everyone would know. So by choosing a section we could plan the birth and make up a believable story as to why we had to go the regional hospital. We were so emotional when she was born, it was unbelievable! I couldn’t take my eyes off her, couldn’t believe this little miracle had come from me. I was advised not to breastfeed which hit me when she was rooting around and when my milk came in, but that was ok as I knew in this case the bottle was best for baby. 

Most of the staff that we have met have been brilliant, but yeah, there have been a few occasions when I have sensed some were whispering about me and I know that one midwife told other midwives about my HIV when they weren’t even looking after me. That hurt. When they came to me with gloves on I wondered was that because of my HIV or do they do that with everyone now? The postnatal ward was an anxious time, with visitors coming in, we were afraid some staff would mention the baby’s medication, or my medication in front of them. It was just so stressful. So many different staff with access to my notes, what if someone let it slip, or another patient overheard, it’s such a small country and someone always knows someone who knows you. We felt really vulnerable—it is like you have done something really bad and you are just waiting to get caught. I was actually glad when I got home. 

Most people still don’t understand about HIV. Some people if they know, don’t want to come near you in case they get it…as if! Like even John who I have been with for 10 years is negative so how likely is it for an acquaintance to get it! 

**John:** We are really enjoying being parents, our baby has been such a blessing, she was started on medication for 4 weeks after delivery and she has tested negative all along and although they were fairly sure we were so relieved when she was confirmed HIV negative after 18 months. And now no more blood tests and finally we can be like any other family. 

## 5. Stage 4: eLearning Production

### 5.1. Methods

The research team worked with a professional production team which included an eLearning educational web developer, a graphic designer and a communication company who were responsible for the casting of actors, filming and final editing of the videos. The research team provided the communication company with character profiles for the actors which reflected the demographics and ethnic diversity of participants in our primary research. Screen tests were taken and viewed before finally deciding on the actor for each vignette. Among the three male and five female actors, three were African, one Asian, one eastern European and three White (from northern Ireland). The healthcare professionals interviewed included, a HIV physician, an obstetrician and two midwives, one of whom was an antenatal coordinator. While our hope was to produce a resource that could have international relevance, our primary focus to produce a resource for Northern Ireland’s maternity care staff influenced our decision to use two Northern Irish actors as the characters in our “whole story” vignette. This decision reflected a desire not only to engage our home audience but also to dispel the myth that HIV is not an issue for heterosexuals from Northern Ireland. In addition, Kate’s image is used in corporate and promotional materials, including a Quick Response (QR) code on the poster, which facilitates direct access to the home page and whole story via a mobile phone. We have encouraged health care professionals to place posters in prominent public places within their services as a means of promoting pregnancy as a normal and achievable goal for HIV affected couples, thereby increasing public awareness and challenging assumptions that an HIV positive woman or man could not or should not conceive.

Filming of the vignettes, “one woman’s story” and interviews with professionals took place in various locations over one week. Each of the eight actors had a discreet vignette to record and then also shorter, engaging quotes which were designed to illustrate elements of the *Living with HIV* module, such as confidentiality, stigma, fatherhood and culturally sensitive care. The result was a total of 26 video clips of eight actors and four healthcare professionals, ranging from 30 Sec to 8 min in length. Only one vignette had to be re- recorded with a different actor, due to a lack of perceived credibility of the first recorded piece. While the communication company was responsible for the production and filming, one member of the research team was always available on location to provide comment on issues of authenticity. In addition, a further day spent in the editing suite provided an opportunity to comment on joint editing decisions. Following the final edit, we worked with the tool developer to place the videos within the resource. What we didn’t expect was the huge volume of text we were able to delete from the resource because, in effect, the videos told most of the story.

### 5.2. Results

“HIV & Pregnancy—Prepared to Care”, which is accessible across all platforms, including mobile phone, is presented in four sections; eLearning resource, Original research, Project development and One woman’s own story. The resource went live and was launched on 29 November 2013 to mark World AIDS Day. Key personnel from Health and Social Care Trusts and other relevant organisations across Northern Ireland were invited, including staff and managers from midwifery, sexual health and education. The event was opened with a guest speech by the Chief Medical Officer from the Department of Health in Northern Ireland. The resource was presented at seven events across the five Health and Social Care Trusts in Northern Ireland between February and March 2014. A total of 188 healthcare professionals attended the events which were promoted mainly within maternity units. Feedback was very positive and subsequently, a link to the resource is now on three Health and Social Care Trust intranets and is promoted as part a programme of continuing professional development. The website can be accessed at this address: www.qub.ac.uk/hivandpregnancy.

## 6. Discussion

Our study was one of the first qualitative studies to use a repeat interview method to specifically explore the perspectives of HIV affected pregnant women and their partners on the care they received from the pre conception to post natal period. The inclusion of men was also a significant contribution to the literature. Previous research had focused on the reproductive desires and intentions of HIV affected women [14,15,16,17,18], their experience of motherhood [19,20,21,22,23] and their concerns about the effect of antiretroviral therapy on themselves and their babies [19,24,25]. This project, funded by a Knowledge Transfer grant from the Health & Social Care Research and Development division at the Public Health Agency for northern Ireland, sought to translate the qualitative evidence from our research into educational material for maternity care practice in Northern Ireland and internationally. Importantly, our research uncovered the importance to patients of the ability of clinicians to perceive and interpret the subjective experiences of HIV positive women and men in their care as being equally important as their level of medical knowledge. While the narrative of HIV treatment is hugely encouraging, with the success of antiretroviral therapy transforming pregnancy into a normal and achievable goal for HIV affected couples, the narrative of the lived experience of HIV continues to be largely overshadowed by a perception of stigma. In their scoping review of arts based health research Boydell *et al*. [26] recognise that the arts and qualitative enquiry share an interest in the significance of the subjective nature of human experience and providing rich description. Hence, the rationale for using an educational approach which would transcend the traditional biomedical approach of focusing on disease treatment by placing the focus primarily on the personal narratives of patient and health professional experience. The interactive eLearning resource described here incorporates key medical information, but the innovative feature of this project, was the way in which precedence was afforded to the qualitative findings of our study which bring to life patient and partner experiences and what it means for nurses, midwives and doctors to be prepared to care for couples affected by HIV. Creative approaches to disseminating health research and narratives in particular, have the potential to reach more diverse audiences and focus on the experiential aspects of reproductive decision making in the context of HIV, communicating the poignancy of patient experiences [11], and evoking an emotional and affective response [27].

This paper provides insights into the process of creating knowledge translation from qualitative research on HIV maternity care. In doing so, we highlight the importance of consultation and gathering the right people around the project team as central to a successful outcome. As the researchers, our responsibility was to safeguard the faithfulness of the artistic representations to the fidelity of our primary research findings. However, as other authors have noted [28], balancing the artisitic and scientific elements of the project can present a significant challenge. In reality, the meetings with consultation groups ensured that resource development also took cognisance of the views of HIV positive women, the professional end users and the service leaders who could support implementation of the product. Equally, discussions with all the skilled professionals in the production team made clear our intention was to produce a resource which aimed to promote greater consciousness among healthcare staff of the significance of stigma in HIV positive men’s and women’s perceptions and experience of care, while also acknowledging that the consequences of HIV have been greatly transformed by developments in antiretroviral therapy.

Our engagement in the process of knowledge translation highlighted a number of limitations. First, this educational resource has been primarily developed with skills in northern Ireland, since healthcare in the United Kingdom is devolved to the four regions. Further internationalisation of this work would benefit from broader partnerships with other nations. Second, while the resource uniquely also includes men as reproductive partners, further research might bring their experiences more to the fore. Third, the evaluation we have to date is solely based on web usage. Further research, which we have planned, using evaluation methods, will increase knowledge in relation to impact on health care professionals’ knowledge and attitudes and health service practice. However, the benefit of this paper is that it makes space for describing the process, which is a key deficit in the knowledge translation literature.

## 7. Conclusions

Overall, our experience confirms the value of qualitative research in knowledge translation and its contribution to evidence based practice. In so doing, we sought to challenge the inequity between the dominance of biomedical knowledge over understanding the patient experience in the preparation of health professionals to care for HIV affected women and men who are having a baby or seeking to have a baby. An arts based educational approach has the potential to encourage the development of self-awareness which prompts the practitioner to be more conscious of how their communication and interactions with people in their care facilitates or undermines the development of a therapeutic partnership.

## References

[1] Graham I. Forward, CIHR Knowledge to Action: An End of Grant Knowledge Translation Casebook. www.chir-irsc.gc.ca/e/documents/cihr.

[2] Sandelowski M., Trimble F., Woodard E., Barroso J. (2006). From synthesis to script: transforming qualitative research findings for use in practice. Qual. Health Res..

[3] Lohan M., Olivari M.G., Corkindale C., Milani L., Confalonieri E., Cruise S., O’Halloran P., Alderdice F., Hyde A. (2013). Adolescent men’s pregnancy resolution choices in relation to an unintended pregnancy: A comparative analysis of adolescent men in three countries. J. Fam. Iss..

[4] Oliffe J.L., Han C., Lohan M., Bottorff J. (2014). Repackaging prostate cancer support group research findings. Am. J. Mens Health.

[5] Barbour R. (2000). The role of qualitative research in broadening the “evidence base” for clinical practice. J. Eval. Clin. Pract..

[6] Kelly C., Lohan M., Alderdice F., Spence D. (2011). Negotiation of risk in sexual relationships and reproductive decision-making amongst HIV sero-different couples. Cult. Health Sex..

[7] Kelly C., Alderdice F., Lohan M., Spence D. (2012). Creating continuity out of the disruption of a diagnosis of HIV during pregnancy. J. Clin. Nurs..

[8] Kelly C., Alderdice F., Lohan M., Spence D. (2013). “Every pregnant woman needs a midwife”—The experiences of HIV affected women in northern Ireland. Midwifery.

[9] British HIV Association Guidelines for the Management of HIV Infection in Pregnant Women. www.bhiva.org/documents/Guidelines/Pregnancy/2012/hiv1030_6.pdf.

[10] Parsons J., Heus L., Moravac C. (2013). Seeing voices of health disparity: Evaluating arts projects as influence processes. Eval. Program Plann..

[11] Blickem C., Priyadharshini E. (2007). Patient narratives: The potential for “patient-centred care” interprofessional learning?. J. Interprof. Care.

[12] British HIV Association (BHIVA) and the Expert Advisory Group on AIDS (EAGA) Position Statement on the Use of Antiretroviral Therapy to Reduce HIV Transmission. www.bhiva.org/documents/Publications/A-Statement-on-the-use-of-antiretroviral-therapy-for-prevention-of-HIV-transmission-Complete-4-21012013.pdf.

[13] Vernazza P., Hirschel B., Bernasconi E., Flepp M. Les Personnes Seropositives ne Souffrant D’aucune Autre MST et Suivant un Traitmant Antiretroviral Efficace ne Transmettent pas le VIH par voix Sexuelle. www.edwinjbernard.com/pdfs/Swiss%20Commission%20statement_May%202008_translation%20EN.pdf.

[14] Nattabi B., Li J., Thompson S.C., Orach C.G., Earnest J. (2009). A systematic review of factors influencing fertility desires and intentions among people living with HIV/AIDS: Implications for policy and service delivery. AIDS Behav..

[15] Barnes D.B., Murphy S. (2009). Reproductive decisions for women with HIV: Motherhood’s role in envisioning a future. Qual. Health Res..

[16] Cooper D., Harris J., Myer L., Orner P., Bracken H. (2012). “Life is still going on”: Reproductive intentions among HIV positive women and men in South Africa. Soc. Sci. Med..

[17] Kirshenbaum S., Hirky E., Correale J., Goldstein R., Johnson M., Rotheram-Borum M.J., Ehrhardt A. (2004). Throwing the dice :Pregnancy decision-making among HIV-positive women in four U.S. cities. Perspect. Sex. Reprod. Health.

[18] Chen J.L., Phillips K., Kanouse D., Collins R., Miu A. (2001). Fertility desires and intentions of HIV positive men and women. Fam. Planning Perspect..

[19] Giles M., Hellard M., Lewin S., O’Brien M. (2009). The “work” of women when considering and using interventions to reduce mother-to-child transmission (MTCT) of HIV. AIDS Care.

[20] Sanders L. (2008). Women’s voices: The lived experience of pregnancy and motherhood after diagnosis with HIV. J. Assoc. Nurses AIDS Care.

[21] Wilson S. (2007). “When you have children, you’re obliged to live”: Motherhood, chronic illness and biographical disruption. Sociol. Health Ill..

[22] Nelms T.P. (2005). Burden: The phenomenon of mothering with HIV. J. Assoc. Nurses AIDS Care.

[23] Sandelowski M., Barroso J. (2003). Towards a metasynthesis of qualitative findings on motherhood in HIV-positive women. Res. Nurs. Health.

[24] Shannon M., Powell Kennedy H., Humphreys J. (2008). HIV-infected mothers’ foci of concern during the viral testing of their infants. J. Assoc. Nurses AIDS Care.

[25] Sherr L., Barnes J., Johnson M. (2000). HIV interventions in pregnancy: The views of HIV positive women. Clin. Psychol. Psychother..

[26] Boydell K.M., Gladstone B., Volpe T., Allemang B., Stasiulis E. (2012). The production and dissemination of knowledge: A scoping review of arts-based health research. Forum Qual. Soc. Res..

[27] Parsons J., Boydell K. (2012). Arts-based research and knowledge translation: Some key concerns for health-care professionals. J. Interprof. Care.

[28] Rossiter K., Gray J., Kontos P., Keightley M., Cplantonia A., Gilbert J. (2008). From page to stage—Dramaturgy and the art if interdisciplinary translation. J. Health Psychol..

